# Epigenome-wide association study of triglyceride postprandial responses to a high-fat dietary challenge[S]

**DOI:** 10.1194/jlr.M069948

**Published:** 2016-11-28

**Authors:** Chao-Qiang Lai, Mary K. Wojczynski, Laurence D. Parnell, Bertha A. Hidalgo, Marguerite Ryan Irvin, Stella Aslibekyan, Michael A. Province, Devin M. Absher, Donna K. Arnett, José M. Ordovás

**Affiliations:** USDA Agricultural Research Service,* Jean Mayer USDA Human Nutrition Research Center on Aging at Tufts University, Boston, MA; Nutrition and Genomics Laboratory,§§ Jean Mayer USDA Human Nutrition Research Center on Aging at Tufts University, Boston, MA; Department of Genetics,† Washington University School of Medicine, St. Louis, MO; Department of Epidemiology,§ School of Public Health, University of Alabama, Birmingham, AL; Hudson Alpha Institute for Biotechnology,** Huntsville, AL; College of Public Health,†† University of Kentucky, Lexington, KY

**Keywords:** DNA methylation, apolipoproteins, atherosclerosis, diet and dietary lipids, lipoproteins, triglycerides, dietary fat, postprandial lipemia

## Abstract

Postprandial lipemia (PPL), the increased plasma TG concentration after consuming a high-fat meal, is an independent risk factor for CVD. Individual responses to a meal high in fat vary greatly, depending on genetic and lifestyle factors. However, only a few loci have been associated with TG-PPL response. Heritable epigenomic changes may be significant contributors to the unexplained inter-individual PPL variability. We conducted an epigenome-wide association study on 979 subjects with DNA methylation measured from CD4^+^ T cells, who were challenged with a high-fat meal as a part of the Genetics of Lipid Lowering Drugs and Diet Network study. Eight methylation sites encompassing five genes, *LPP*, *CPT1A*, *APOA5*, *SREBF1*, and *ABCG1*, were significantly associated with PPL response at an epigenome-wide level (*P* < 1.1 × 10^−7^), but no methylation site reached epigenome-wide significance after adjusting for baseline TG levels. Higher methylation at *LPP*, *APOA5*, *SREBF1*, and *ABCG1*, and lower methylation at *CPT1A* methylation were correlated with an increased TG-PPL response. These PPL-associated methylation sites, also correlated with fasting TG, account for a substantially greater amount of phenotypic variance (14.9%) in PPL and fasting TG (16.3%) when compared with the genetic contribution of loci identified by our previous genome-wide association study (4.5%). In summary, the epigenome is a large contributor to the variation in PPL, and this has the potential to be used to modulate PPL and reduce CVD.

Postprandial lipemia (PPL) refers to the changes in plasma lipoproteins following food consumption. PPL is highly correlated with fasting plasma TG concentrations, yet some evidence suggests that PPL may be an independent risk factor for CVD (1–4) in ways distinct from fasting TG (5–7). The risk of CVD that can be independently attributed to elevated or elongated PPL (factors relating to amount and time) is supported by the fact that modern humans spend most of their lifetimes in the postprandial state (2). A persistent and elevated presence of TG-rich lipoprotein (TRL) particles in the circulation promotes thrombotic processes, which augment the risk for CVD events (3). PPL varies greatly among individuals, being defined, in addition to the characteristics of the food ingested, by age, sex, genetic variation, and environmental exposures (1, 3). Several genome-wide association studies (GWASs) for fasting TG have been performed, revealing over 30 related loci (8, 9). However, only two GWASs have been conducted for PPL-TG (10, 11), and only four genetic variants, which are also associated with fasting TG, have been identified in relation to PPL (9–12). This may result from the limited sample sizes of these studies combined with the strong environmental influence on this phenotype (13).

Environmental factors exert significant effects on gene expression, in some instances through epigenetic mechanisms (14). Thus, epigenetic marks can be considered “fingerprints” of that communication between the environment and the genome and some experimental evidence indicates that diet-induced epigenetic changes can be transmitted through several generations (15, 16). As such, the environment, including habitual diet, may contribute to the health status of the individual and his/her descendants (17).

We hypothesize that individuals respond to environmental exposures by modulation of the epigenome, which elicits changes in the PPL response that could alter CVD risk. Of different forms of epigenetic modification, DNA methylation is the most extensively studied for its technical feasibility at the epigenome-wide scale, cost-effectiveness, well-established standard analysis platform, and its apparent relation to nutrition (18). The objective of this study was to conduct an epigenome-wide association analysis in order to identify DNA methylation sites that were associated with PPL-TG concentrations in response to a high-fat meal in the Genetics of Lipid Lowering Drugs and Diet Network (GOLDN) study. In addition, we have characterized DNA sequence variation in the significant epigenome-wide association study (EWAS) regions in relation to PPL-TG responses.

## MATERIALS AND METHODS

### Study population

The GOLDN study, as a part of the National Heart, Lung, and Blood Institute Family Heart Study, recruited participants (n = 1,327) from families of European descent at two field centers: Minneapolis, MN and Salt Lake City, UT. GOLDN was designed as an intervention study to identify genetic factors that determine lipid responses to two interventions: *1*) a high-fat meal test; and *2*) a 3 week treatment of fenofibrate (160 mg). Participants were requested to stop the use of lipid-lowering medication for at least 4 weeks and to refrain from alcohol for 24 h prior to their study visit. Diet history questionnaires were used to collect demographic, lifestyle, and dietary data (19). The study protocol was approved by the Institutional Review Boards at Tufts University, the University of Minnesota, the University of Utah, and the University of Alabama at Birmingham. All participants provided written consent for the study. The current study comprised a total of 979 participants for whom complete PPL and epigenome data exist.

### Postprandial phenotypes

Postprandial TG responses were calculated based on the growth curve models of TG as the function of times, as described (10). Briefly, the postprandial phenotypes were estimated as four measurements: uptake, clearance, area under the whole curve (AUC), and area under the curve increase (AUI). Uptake was estimated as the slope of the TG response from 0 to 3.5 h after the meal consumption, a time at which most fat from the meal has been absorbed (3). Clearance was defined as the downward slope of the TG level from 3.5 to 6 h after meal consumption, which measures the speed of the metabolic process that metabolizes the excess fat from the plasma (3, 20). The AUC was calculated as the total AUC according to the trapezoid method, and the AUI was estimated by subtracting the baseline area from the AUC (10).

### Epigenome analysis

Different cell types in whole blood may have contrasting methylation patterns. Thus, to minimize the confounding effect of cell type differences and increase the consistency of methylation measures across samples, we restricted DNA methylome analysis to CD4^+^ T cells, which represent the most common lymphocytes in whole blood (21, 22). Using CD4^+^ specific antigen magnetic beads (Invitrogen, Carlsbad, CA), CD4^+^ T cells were isolated from frozen buffy coat samples that were collected at visit 2 (baseline) before fenofibrate intervention. DNA was extracted from these cells using DNeasy kits (Qiagen, Venlo, The Netherlands) (23). Genome-wide DNA methylation of all DNA samples was quantified as described (23) using Illumina Infinium human methylation 450K arrays (Illumina, San Diego, CA), which contain over 485,000 probe sets to measure DNA methylation of over 450,000 CpG sites across the human genome. Using Illumina’s GenomeStudio package, we estimated the proportion of total signal of methylation for each probe as the β score, and detection *P* value as the probability that the total intensity for a given probe falls within the background signal intensity. Methylation signals were then further filtered out if CpG sites met one of the following criteria: *1*) detection *P* > 0.01 and 1.5% of samples have missing data; or *2*) >10% of samples have no adequate intensity (23). For adjustment of the batch effect across samples, the filtered β scores were normalized separately for Infinium I and II probe sets using the ComBat package for R (22, 24, 25). At the end, 464,005 CpG sites passed quality control and these were used for statistical analysis in this study. To control for heterogeneity of CD4^+^ T cells across all samples, principal components (PCs) based on the β scores of all autosomal CpG sites that passed quality control were calculated using the prcomp function in R (v12.12.1). Four PCs were used in the EWAS.

### Genome-wide genotyping

The detailed procedure of genome-wide genotyping in GOLDN has been described (10, 26). In this study, we used the hybrid genotype data of 2,543,887 SNPs, among which 484,029 were genotyped using the Affymetrix Genome-wide 6.0 Array (Affymetrix, Santa Clara, CA). The remaining SNPs were imputed using MaCH software (version 1.0.16) with human genome build 36 as reference, and genotyped SNPs that met the following criteria (27): call rate >96%, minor allele frequency >1%, and Hardy-Weinberg equilibrium test *P* > 10^−6^.

### Statistical methods

#### Data analysis design.

As the epigenome is known as the fingerprint of individuals in response to lifetime exposures up to the time point when the samples were collected, an individual epigenotype depends on local environments. Therefore, individuals with the same genotype may have different epigenotypes under different environments. For such reasons, the best discovery and replication in EWASs should be done within the same population. We randomly split the whole population (n = 979) into two-thirds as a discovery sample (n = 653) and one-third as a replicate sample (n = 326) using Proc Surveyselect in SAS v9.4 (Cary, NC) while holding the distributions of baseline TG, BMI, and sex similar between the two samples. To examine the differences in clinical characteristics between the sexes among the discovery and replication, we performed a *t*-test.

#### Epigenome-wide association.

In the discovery stage, we modeled the association between methylation β score at each CpG site and PPL response measures using a linear mixed model (28), adjusting for sex, age, age^2^, age^3^, study site, and the first four PCs for T cell impurity as fixed effects, and kinship as a random effect. The kinship matrix was generated based on family pedigree (29). The analysis was implemented in SNP and VARIATION SUITE 8.4.3 (GoldenHelix Inc., Bozeman, MT). In addition, we conducted an EWAS adjusted for an additional covariate of baseline TG. We applied the Bonferroni correction, setting epigenome-wide significance at 1.1 × 10^−7^ (25). We subsequently fitted the identical model in the replication sample (n = 326) for the CpG sites that were statistically significant in the discovery set for PPL measures. We corrected the threshold for significance in the replication stage for multiple testing using the Bonferroni approach, *P* = 0.05/number of replicated sites. Applying the concept of meta-analysis, we then combined the discovery and replication samples (i.e., the entire sample n = 979) and repeated the analysis using the same models (with or without adjusting for the baseline TG) as in the discovery and replication stages. Because PPL-TG response traits are strongly correlated with fasting TG, we also conducted an EWAS for fasting TG with the entire population using the same method and model (without adjusting for baseline TG).

### Estimation of genetic and epigenetic variance contribution

Variance contribution of individual methylation sites was estimated using efficient mixed-model association while controlling for normalized kinship (28) that was calculated based on family pedigree (29). This procedure was implemented in the Mixed Linear Model Analysis tools of SNP and VARIATION SUITE 8.4.3 (GoldenHelix Inc., Bozeman, MT). As the identified methylation sites were not totally independent from each other, the combined variance contribution of all methylation sites was estimated with the option of Multi-Locus Mixed Model of the Mixed Linear Model Analysis while controlling for family relationship and covariates. The variance contribution of the previously identified genetic variants (rs964184 and rs10243693) that were associated with AUC (10) was calculated using the same method in participants (n = 707) for whom the genotype data was available.

### Relationship between epigenetic markers and genetic variants

For the CpG sites that showed a significant association, we further examined their correlations with loci previously identified (10) that were associated with AUC in participants for whom both epigenome and genome data were available (n = 707). In addition, we further examined the association of SNPs within a 50 kb region of each CpG site associating with AUC. Data from previous genetic association studies were retrieved from the GWAS catalog (30) and gene-environment interactions from CardioGxE (31).

## RESULTS

### Demographic and clinical characteristics

The TG-related characteristics of the discovery and replication samples are listed in **Table 1**. There were no significant differences between the discovery and replication samples for the TG and PPL response-related phenotypes (Table 1). However, there were equivalent significant differences in the baseline TG and TG AUC between sexes within each sample.

**TABLE 1.** Characteristics of discovery and replicate samples in GOLDN

	Discovery Sample (n = 653)			Replication Sample (n = 326)		
	Men	Women	Both	Men	Women	Both
n	313	340	653	156	170	326
Age, years	48.1 (15.9)	47.9 (16.4)	48.0 (16.2)	50.2 (17.3)	47.6 (16.4)	48.8 (16.9)
BMI, kg/m^2^	28.3 (4.6)	28.2 (6.4)	28.2 (5.6)	28.5 (5.0)	28.2 (6.4)	28.3 (5.8)
Waist, inches	100.0 (13.8)	93.2 (17.8)	96.5 (16.4)	100.6 (13.6)	93.1 (16.9)	96.7 (15.8)
TG at baseline, mg/dl	149.1 (111.0)*^a^*	125.7 (82.2)	136.9 (97.7)	144.4 (90.1)	127.2 (87.3)	135.4 (89.0)
TG uptake slope	0.18 (0.03)	0.17 (0.03)	0.18 (0.03)	0.18 (0.03)	0.18 (0.03)	0.18 (0.03)
TG clearance slope	−0.06 (0.05)	−0.07 (0.05)	−0.07 (0.05)	−0.05 (0.05)	−0.07 (0.05)	−0.06 (0.05)
TG AUC	31.6 (3.4)*^b^*	30.4 (3.3)	30.9 (3.4)	31.6 (3.3)*^a^*	30.3 (3.3)	30.9 (3.4)
TG AUI	2.5 (0.5)	2.4 (0.5)	2.4 (0.6)	2.6 (0.6)	2.4 (0.5)	2.5 (0.6)

Values are means (standard deviations).

a *P* value for differences between men and women (within sample) significant with *P* < 0.05.

b *P* value for differences between men and women (within sample) significant with *P* < 0.001.

### Epigenome-wide association of PPL

We first conducted epigenome-wide association tests for each of four PPL-TG traits (**Table 2**) in the discovery sample (n = 653). For AUC, we identified four methylation sites in three genes (*CPT1A*, *APOA5*, and *SREBF1*) that reached epigenome-wide significance at *P* ≤ 1.1 × 10^−7^ (Table 2). However, when adjusted for baseline TG, no methylation site reached epigenome-wide significance (supplemental Table S1). For the other three TG response traits (uptake, clearance, and AUI), we did not find any methylation sites that reached epigenome-wide significance either with or without adjusting for baseline TG. We then replicated the findings from the discovery stage in the replication sample (n = 326; Table 2). All four sites that were associated with AUC in the discovery sample replicated in the second sample after correction for multiple testing (*P* < 0.05/4 = 0.0125). After adjustment for baseline TG, only two methylation sites (cg00574958 and cg17058475 at *CPT1A*) were replicated in the replication sample (supplemental Table S1).

**TABLE 2.** CpG sites associated with AUC in response to a high-fat meal in discovery and replication samples

Marker	Chr:Position*^a^*	Gene	Discovery (n = 653)		Replication (n = 326)	
			β (SE)	*P*	β (SE)	*P*
cg00574958	11:68607622	*CPT1A*	−33.24 (4.91)	3.02 × 10^−11^	−50.84 (6.27)	1.18 × 10^−14^
cg17058475	11:68607737	*CPT1A*	−19.93 (3.57)	3.58 × 10^−8^	−33.53 (5.01)	1.01 × 10^−10^
cg12556569	11:116664039	*APOA5*	3.41 (0.63)	9.52 × 10^−8^	2.61 (0.92)	4.97 × 10^−3^
cg11024682	17:17730094	*SREBF1*	25.41 (4.64)	6.10 × 10^−8^	16.77 (6.03)	5.74 × 10^−3^

a Genomic position was based on genome build 37.

**Fig. 1.**
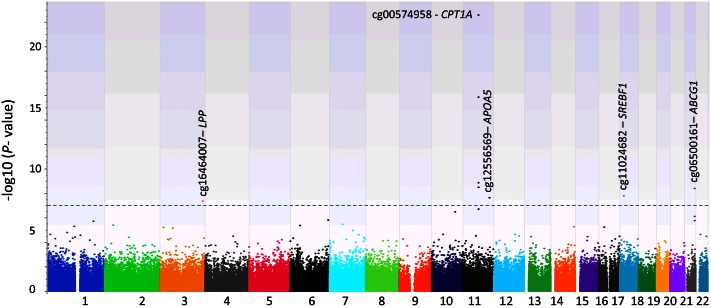
Distribution of *P* values [−log10(*P* value)] from the epigenome-wide association analysis with AUC phenotype (n = 979). Eight CpG sites reached epigenome-wide significance *P* < 1.1 × 10^−7^ (above the dashed line).

Applying the concept of meta-analysis, we next conducted a third epigenome-wide association analysis by combining the discovery and replication samples and using the same models (**Table 3**). From this analysis, we observed eight methylation sites that were associated with AUC at the level of epigenome-wide significance (at *P* ≤ 1.1 × 10^−7^; see Table 3, **Fig. 1**, supplemental Fig. S1). Four of these eight CpG sites were identified in the initial analysis (Table 2). The four newly identified sites were in the *CPT1A*, *LPP*, and *ABCG1* genes. These four sites were also significantly associated with AUC in the discovery and replication samples (supplemental Table S2), with *P* values ranging from 3.39 × 10^−4^ to 1.05 × 10^−7^. At *CPT1A*, four CpG sites were highly correlated with each other, with correlation coefficients ranging from 0.643 to 0.843 (supplemental Table S3) and similar associations with the AUC. Thus, these four CpG sites, located at the promotor region of *CPT1A*, likely represent one methylation region at *CPT1A*. Interestingly, three other methylation sites at *LPP*, *SREBF1*, and *ABCG1* are also correlated with these four *CPT1A* methylation sites (supplemental Table S3). In contrast, cg12556569 at *APOA5* was independent of all seven methylation sites, but when adjusting for baseline TG, no methylation site reached epigenome-wide significance. Lastly, there was no significant association between methylation sites and the other three PPL-TG measures of uptake, clearance, and AUI.

**TABLE 3.** CpG sites associated with AUC in response to a high-fat meal in the full samples (n = 979)

Marker	Chr:Position*^a^*	Gene	β (SE)	*P*	AUC Variance Explained
cg16464007	3:188002729	*LPP*	12.81 (2.32)	4.50 × 10^−8^	0.030
cg00574958	11:68607622	*CPT1A*	−38.50 (3.77)	2.69 × 10^−23^	0.097
cg09737197	11:68607675	*CPT1A*	−16.79 (2.75)	1.39 × 10^−9^	0.037
cg17058475	11:68607737	*CPT1A*	−23.86 (2.84)	1.39 × 10^−16^	0.068
cg01082498	11:68608225	*CPT1A*	−43.83 (7.34)	3.33 × 10^−9^	0.036
cg12556569	11:116664039	*APOA5*	2.94 (0.52)	2.30 × 10^−8^	0.032
cg11024682	17:17730094	*SREBF1*	20.64 (3.63)	1.68 × 10^−8^	0.032
cg06500161	21:43656587	*ABCG1*	16.59 (2.80)	4.25 × 10^−9^	0.035

a Genomic position was based on genome build 37.

In this population, we found that AUC was strongly correlated with fasting TG (Pearson correlation coefficient *r* = 0.853; supplemental Table S6). We then conducted an EWAS for fasting TG with the entire population (n = 979) using the same method, and found six of eight AUC-associated methylation sites were significantly associated with fasting TG at the epigenome-wide significance (supplemental Table S4), and two other sites (cg12556569 and cg11024682) at *APOA5* and *SREBF1* almost reached the epigenome-wide significance (*P* = 1.47 × 10^−7^ and 1.66 × 10^−7^, respectively).

### Phenotypic variation of PPL explained by the eight identified methylation sites

To measure how much variation in AUC can be accounted for by identified epigenetic variants, we estimated the variance contribution of each significant CpG site to the phenotypic variance of AUC (Table 3). In particular, cg00574958 had the largest effect on AUC (9.7%), whereas cg16464007 at *LPP* had the smallest contribution of 3.0%. Four CpG sites at *CPT1A* contributed a similarly large amount of variance to PPL phenotype. As all CpG sites, except cg12556569 at *APOA5,* are not totally independent from each other (supplemental Table S3), we estimated the combined variance contribution of all eight CpG sites together. This yielded a value of 14.9% for AUC variance. As AUC is strongly correlated with fasting TG, we also estimated the combined variance contribution of the eight sites to the phenotypic variance of fasting TG and determined this to be 16.3%. In contrast, the genetic variance contribution of the two previously identified genetic loci (rs964184 and rs10243693) (10) was estimated as 4.5% both for AUC and fasting TG, thereby suggesting that the contribution of epigenetic variants identified in this study to AUC and fasting TG is substantial in this population.

### Correlation between identified methylation sites and PPL-associated variants

**Fig. 2.**
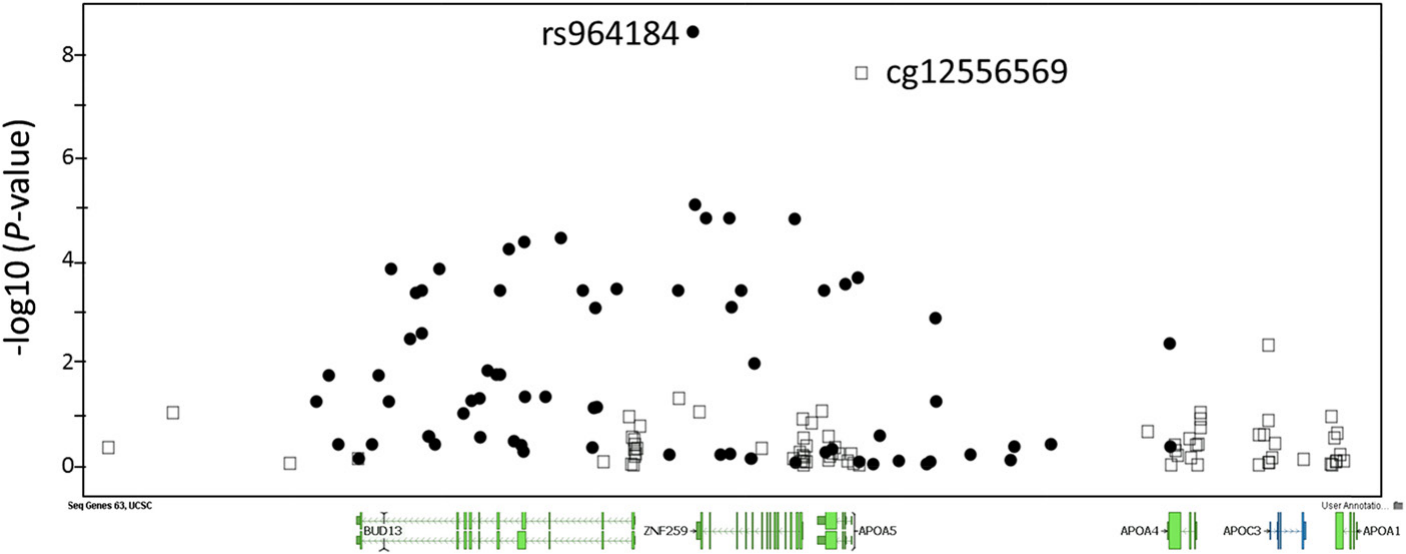
Integrated regional overlap of EWAS signals (open squares) and GWAS signals (solid circles) at the *APOA5*. The x-axis displays the physical position of CpG sites and SNPs within 50 kb upstream and downstream of the EWAS signal of cg12556569 at *APOA5*; the y-axis displays −log10(*P* value) of the association.

We examined the correlation between the eight identified methylation sites and two genetic variants (rs964184 and rs10243693) that we previously found in GOLDN to be associated with AUC (10). The methylation level at cg12556569 at *APOA5* was highly correlated (*r* = −0.689, *P* = 2.71 × 10^−100^) with the genotype of SNP rs964184 (supplemental Table S4). However, methylation levels at any of the identified CpG sites were not associated with the rs10243693 genotype. In addition, we also examined all SNPs within 50 kb upstream or downstream of each of the eight methylation sites for association with AUC. For cg12556569 at *APOA5*, there were 20 SNPs (of 71 SNPs in this region; **Fig. 2**, supplemental Table S5) that reached significant association after correction for multiple testing (*P* ≤ 0.05/71 = 0.0007). For the other seven methylation sites, none of the SNPs evaluated reached significant association after correction for multiple testing.

## DISCUSSION

AUC measures the total change in plasma TG (PPL) and the duration of these changes after consumption of a high-fat meal (10). In terms of the risk for CVD, an increased AUC indicates elevated levels of atherogenic TRLs and/or an extended period of time that such lipoproteins remain in the circulation (2, 3, 5). Our findings highlight the significant contribution of the epigenome to the individual variability in PPL response to a high-fat challenge. PPL is influenced strongly by environmental factors and these environmental factors may convey their effects through epigenetic mechanisms. We identified eight methylation CpG sites that were significantly associated with PPL-TG responses (i.e., AUC) in our study with 979 subjects of European descent. AUC is strongly correlated with fasting TG, and we also observed that these eight methylation sites showed similar strong associations with fasting TG. These methylation sites encompass five genes: *LPP*, *CPT1A*, *APOA5*, *SREBF1*, and *ABCG1*, four of which encode proteins known to be involved in lipid metabolism. Four CpG sites in *CPT1A*, *APOA5*, *SREBF1*, and *ABCG1* were reported recently to be associated with fasting TG in two populations of European descent (KORA and InCHIANTI) (32), lending support to our findings.

Individuals respond diversely to a high-fat meal, with TG reaching the highest peak 3.5–4 h after the meal and returning to baseline at 10 to 12 h after consumption (5). In the GOLDN population, AUC was calculated based on measures at three time points: 0, 3.5, and 6 h after consumption of the 83% fat meal. Previously, our GWAS identified two loci that were associated with AUC, but such associations were no longer significant after adjustment for baseline TG (10). Similarly, in the present study, all eight identified methylation sites did not reach epigenome-wide significance after adjustment for baseline TG (supplemental Table S1). As fasting TG was measured from the same blood draw as PPL in this population, we observed a strong correlation between fasting TG and AUC (*r* = 0.853; supplemental Table S6). Hence, it is not unexpected that identified epigenetic variants that are associated with AUC are also highly correlated with fasting TG, thus contributing to the risk of CVD.

CPT1A converts cytoplasmic long-chain acyl-CoA to acylcarnitine, which is then transported to mitochondria for fatty acid β-oxidation. Decreased methylation status at the *CPT1A* locus has been associated with lipid profile (22, 33), insulin resistance (25), and metabolic syndrome (34), as well as obesity (35) in GOLDN, and some of these findings were replicated in other populations (35). In addition, increased methylation at *CPT1A*-cg00574958 is correlated with decreased expression of *CPT1A* (22, 36) and, more generally, an inverse relation was seen between *CPT1A* expression and changes in TG levels after fish oil supplementation (37). Interestingly, CpG methylation at *CPT1A* was noted as 1.49-fold higher in adipocytes of obese compared with never obese women (38). We identified four correlated CpG sites at *CPT1A* that strongly associated with AUC (supplemental Table S3), and decreased methylation of the same four sites has been associated with increased fasting plasma TG (22). Moreover, obese subjects show increased PPL responses to a high-fat meal (9, 39, 40). As the methylation of *CPT1A* contributes to increased risk of obesity in several populations (35), it is not surprising that *CPT1A* methylation is highly correlated with PPL responses, likely linked to risk of CVD.

The *APOA5* genotype is a strong determinant of fasting TG (41) and PPL-TG (12). We identified SNP rs964184 near *APOA5* as highly associated with PPL-TG (10). In this study, we found that methylation site cg12556569 (Fig. 2), 903 bp from the *APOA5* transcription start site, was significantly associated with the PPL-TG response. This methylation site is highly correlated with three SNPs (see supplemental Table S3): rs964184 (*r* = −0.689), rs662799 (−1131T>C or −600T>C, *r* = 0.480), and rs3135506 (Ser19Trp, S19W or 56C>G, *r* = 0.457). The correlation between cg12556569 and rs964184 was also observed in other populations of European descent (32). Methylation site cg12556569 maps 332 bp upstream of rs662799, a functional variant with a haplotype containing the C allele conferring 46% lower luciferase activity (transcription) of *APOA5* (42). We further showed that higher methylation levels at cg12556569 are correlated with elevated PPL-TG responses (Table 3). Thus, it is anticipated that high methylation at this site could be associated with low expression of *APOA5*, thereby leading to abnormal metabolism and accumulation of TRLs.

*ABCG1*, encoding a member of the ABC transporter family, is involved in the efflux of cholesterol and phospholipids from macrophages into HDL. We observed that higher methylation levels at cg06500161, near the *ABCG1* gene, were correlated with a high PPL-TG response. Previously, high DNA methylation levels at *ABCG1* have been associated with increased fasting TG (32, 43). In addition, high methylation levels at this CpG site were shown to be associated with increased fasting insulin and insulin resistance (25). Furthermore, type 2 diabetes patients showed low expression of *ABCG1* and increased intracellular cholesterol accumulation (44). These observations support the notion that higher methylation of *ABCG1* could lead to an increased CVD risk.

*SREBF1*, a transcription factor that binds to the sterol regulatory element-1, regulates transcription of the LDL receptor gene (44). MicroRNA *MIR33B*, encoded within intron 17 of *SREBF1*, targets cholesterol metabolism and fatty acid oxidation genes (45, 46) leading to increases in VLDL-TG (47) and altered expression of *CPT1A* (48). Interestingly, a gene-diet interaction between *SREBF1* variant rs2297508 (Gly952Gly) and a high-carbohydrate low-fat diet was reported for plasma TG in Han Chinese women (49). Still, it remains unclear whether the epigenetic association observed here exerts impact on *SREBF1*, *MIR33B*, or both, but it is intriguing that this microRNA has been reported to modulate expression of *ABCG1* and *CPT1A* in human liver cells (48, 50).

*LPP*, also known as LIM domain containing preferred translocation partner in lipoma, functions in cell-cell adhesion and cell motility, but there is no report in relation to its potential role in lipid metabolism and PPL responses. The *LPP* locus has been identified as associating with type 2 diabetes susceptibility (51, 52) and obesity (53), with CpG sites found to be age-associated in a set of seven large extended families (54). The CpG site showing association with TG PPL in this study is about 550 kb from *BCL6*, a locus identified via TG pathway analysis of GWAS results, as was *ABCG1* (55). Moreover, we have noted that the *LPP* locus appears to be under positive selection for obesity traits (56), which may reflect selective pressure from environmental exposure.

All eight identified epigenetic loci in this population are associated with the AUC-PPL phenotype, but there is no significant epigenetic locus that is associated with the other three PPL phenotypes: uptake, clearance, and AUI. We have examined the correlation between all four PPL phenotypes and baseline TG (see supplemental Table S6). Only AUC is highly correlated with baseline TG (*r* = 0.853). The other three PPL traits are weakly (*r* = 0.15 for clearance slope), negatively (*r* = −0.10 for uptake slope), or not (*r* = 0.036 for AUI) correlated with baseline TG. On the other hand, as the PPL was limited to 6 h with only 3 measures (0, 3.5, and 6 h), AUC may not reflect the length of time needed to capture the catabolic side of the PPL, and the data points were not dense enough to precisely calculate the absorption and synthesis of TRLs (and their catabolism). Still, AUC may provide a more comprehensive view of the overall PPL response than the other three phenotypes. Additionally, the other three PPL traits (intestinal absorption, chylomicron synthesis, and hepatic TRL catabolism) may not be properly captured with methylation measures in the CD4^+^ T cells. Therefore, the fact that identified epigenetic loci are associated with AUC only, not the other three measures of PPL, could reflect the particular characteristics of epigenetic loci that are induced by the environment.

It is well-established that obese individuals exhibit elevated postprandial TG in response to a high-fat meal compared with nonobese individuals (57, 58), but the underlying mechanism is not clear. Because epigenetic markers can affect both BMI/waist and PPL phenotypes, in our epigenome-wide association analysis, BMI or waist were not adjusted in our linear mixed models. In fact, three of the identified epigenetic loci, *CPT1A*-cg00574958, *SREBF1*-cg11024682, and *ABCG1*-cg06500161, were also associated with BMI in the meta-analysis that was reported recently based on this population and the Framingham Heart Study (35). To examine the dependence of AUC on obesity, we conducted EWASs with the entire sample (both discovery and replicate samples combined) while adjusting for waist or BMI. Indeed, only cg00574958 (and related loci cg17058475 and cg09737197) at *CPT1A* remained significant at the epigenome-wide level (*P* < 1.1 × 10^−7^), while other loci were marginally significant with AUC at *P* < 5.5 × 10^−6^. In essence, our findings imply that the identified epigenetic loci at *CPT1A*, *SREBF1*, and *ABCG1* can link obesity to hyperlipidemia (high TG) and elevated postprandial TG response, and then to the risk of CVD. This observation potentially defines a mechanism by which obese subjects show increased PPL response to a high-fat meal, with a subsequent increased risk for CVD.

Many GWASs have identified genetic loci for a given trait, but often these loci account for only a small fraction of genetic variation and phenotypic variation, raising the issue of missing heritability (59). Within the same population, we previously conducted a GWAS of PPL-TG response and identified two variants that were associated with AUC and reached genome-wide significance (10). However, these loci explain only about 4.5% of the total phenotypic variation of the PPL response. Diet-induced epigenetic variation in obesity has been demonstrated recently and is transmissible in mice from one generation to next (15). Here, we estimated the variance contribution of the identified eight methylation sites to PPL responses and fasting TG to be 14.9 and 16.3%, respectively. Such a large contribution of the epigenome to PPL-TG and fasting TG variation is striking. This observation suggests that epigenetic processes may account for some of the variation that remains unexplained using the GWAS approach. This underscores the need to include epigenetic markers with genomic markers and gene-environment and epistatic interactions in order to gain a more accurate prediction of CVD risk, and to facilitate the development of effective strategies for its prevention.

This study has its limitations in the following aspects. First, one main limitation with the study is the impossibility to infer the causal relationship between observed DNA methylation and plasma lipid profile. On the one hand, DNA methylation of key lipid metabolism genes, such *APOA5*, can lead to reduced expression, and then slow clearance of TG and elevated PPL (12). On the other hand, it was shown statistically that high TG can also lead to methylation of lipid metabolism genes (*CPT1A* and *SREBF1*) (60). However, the biological and molecular mechanisms of such a causal relationship remain to be demonstrated. The second limitation is the constraint of a 6 h timeframe for the PPL measures with only three time points (0, 3.5, and 6 h), which may not precisely capture the entire PPL. An additional limitation of the study is the cell-type specificity and biological relevance of the identified methylation sites to lipid metabolism. The more relevant tissues for lipid metabolism are liver and intestine, which are not available for analysis in a population study. On the other hand, accumulating evidence suggests methylation sites that are age-related are commonly shared across many tissues (61). DNA methylation in the blood can serve as a biomarker of methylation in other tissues (61). As such, our finding translates from one tissue type to another. Another limitation is that the replication sample of this study is not fully independent of the discovery sample. Considering strong dependence of epigenetic changes on environment, replication within the same population is justified. Furthermore, as the AUC of PPL phenotype was measured in this study in response to consumption of a meal high in dairy fat (83%), the identified epigenetic loci are likely specific to such dietary challenges and our results might not be replicated following other diets. Still, the identified loci map to genes with well-characterized functions in lipid metabolism and homeostasis. On the other hand, based on the power calculation, the discovery sample has sufficient power to identify methylation sites that associate with these PPL phenotypes, as does the replication sample to replicate any findings from the discovery sample.

## Supplementary Material

Supplemental Data
