# Supplementary material for: Epigenome-wide association study of triglyceride postprandial responses to a high-fat dietary challenge
Source: J Lipid Res. 2016 Nov 28;57(12):2200–7. doi: 10.1194/jlr.M069948 (PMC5321216; doi:10.1194/jlr.M069948)
Supplement: Supplemental Data [file supp_57_12_2200__index.html]

Epigenome-wide association study of triglyceride postprandial responses to a high-fat dietary challenge — Epigenome-wide association study of triglyceride postprandial responses to a high-fat dietary challenge — Supplemental Data 

# Epigenome-wide association study of triglyceride postprandial responses to a high-fat dietary challenge

## Supplemental Data

- Supplemental Fig 1 and Table S1-S6 (.pdf, 185 KB) - Fig. S1 Quantile-quantile plot (QQ plot) of AUC epigenome-wide association analysis of the full sample (n=979) Table S1. CpG sites associated with AUC in response to a high-fat meal adjusted for baseline TG level 030.010Table S2. Eight CpG sites associated with AUC in responses to a high-fat meal in the discovery, replication, and full samples of GOLDN Table S3. Pearson's correlation coefficients (the first panel)and P-values (the second panel) between eight AUC-associated methylation sites and four SNPs (n=707) Table S4. Eight CpG sites associated with AUC and TG (log10) in response to a high-fat meal in the full samples of GOLDN Table S5. 71 SNPs of the APOA5 region on the chromosome 11 associated with TG postprandial responses (n=707) Table S6. Pearson's correlation coefficients (the left panel) and P-values (the right panel) between fasting triglyceride (at baseline) and postprandial TG response measures (n=979)
