# Supplementary material for: Epigenome-wide association study of triglyceride postprandial responses to a high-fat dietary challenge
Source: J Lipid Res. 2016 Nov 28;57(12):2200–7. doi: 10.1194/jlr.M069948 (PMC5321216; doi:10.1194/jlr.M069948)
Supplement: Supplemental Data [file 10.1194_M069948_jlr.M069948-1.pdf]

Epigenome-wide association study of triglyceride postprandial responses to a high-fat dietary challenge

Chao-Qiang Lai[1*], Mary K. Wojczynski[2], Laurence D. Parnell[1], Bertha A. Hidalgo[3], Marguerite Ryan Irvin[3], Stella Aslibekyan[3], Michael A. Province[2], Devin M. Absher[4], Donna K. Arnett[3], José M. Ordovás[1]

Supplemental materials

Fig. S1 Quantile-quantile plot (QQ plot) of AUC epigenome-wide association analysis of the full sample (n=979)

Figure S1

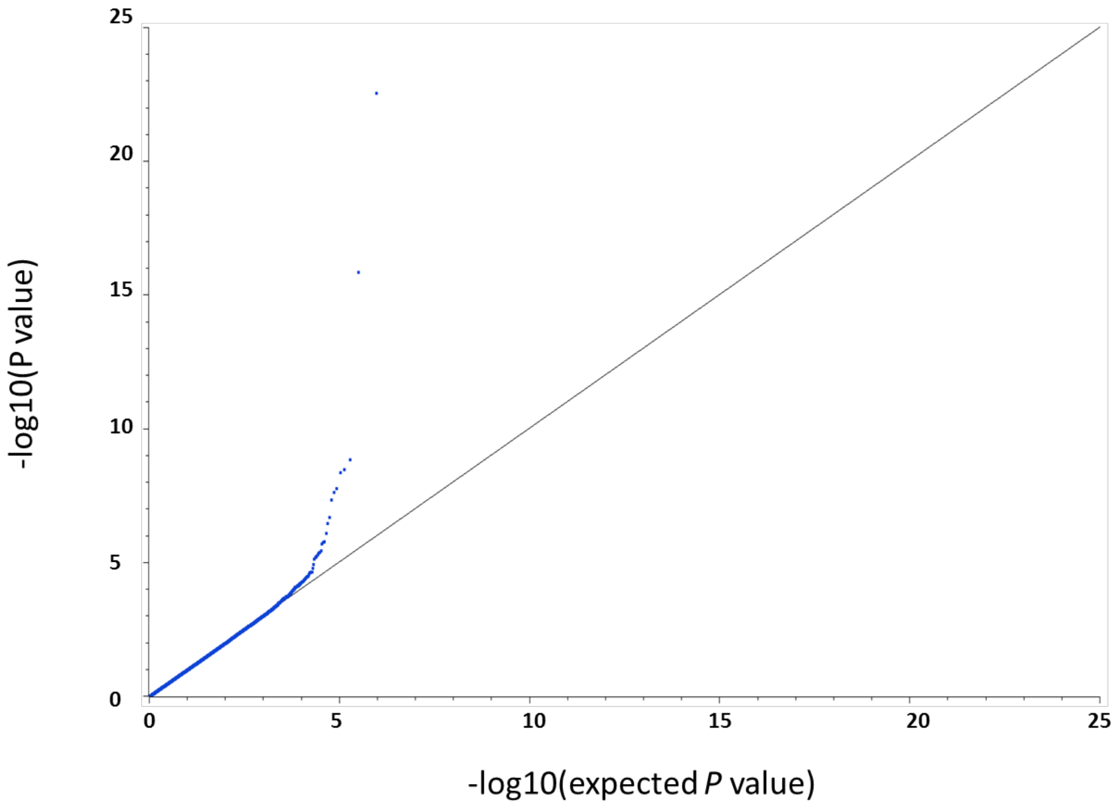

Table S1. CpG sites associated with AUC in response to a high-fat meal adjusted for baseline TG level

			Discovery (n=653)		Replication (n=326)		Full sample (n=979)		
Marker	Chr:position	Gene	beta (se)	*P*-Value	beta (se)	*P*-Value	beta (se)	*P*-Value	AUC variance explained
cg16464007	3:188002729	*LPP*	1.64 (1.65)	3.20E-01	1.58 (2.05)	4.42E-01	1.95 (1.29)	1.32E-01	0.002
**cg00574958**	**11:68607622**	***CPT1A***	**-2.43 (2.96)**	**4.11E-01**	**-13.99 (3.40)**	**5.00E-05**	**-6.99 (2.2.4)**	**2.00E-03**	**0.002**
cg09737197	11:68607675	*CPT1A*	-0.57 (1.98)	7.72E-01	-6.18 (2.40)	1.10E-02	-2.90 (1.54)	5.99E-02	0.004
**cg17058475**	**11:68607737**	***CPT1A***	**-1.18 (2.10)**	**5.73E-01**	**-7.24 (2.63)**	**6.19E-03**	**-3.60 (1.63)**	**2.80E-02**	**0.005**
cg01082498	11:68608225	*CPT1A*	-6.81 (5.35)	2.04E-01	-9.66 (6.37)	1.31E-01	-9.20 (4.09)	2.48E-02	0.005
**cg12556569**	**11:116664039**	***APOA5***	**0.98 (0.36)**	**6.00E-03**	**0.77 (0.44)**	**8.20E-02**	**0.91 (0.281)**	**1.00E-03**	**0.011**
**cg11024682**	**17:17730094**	***SREBF1***	**8.09 (2.64)**	**2.00E-03**	**2.46 (2.91)**	**3.98E-01**	**5.60 (2.00)**	**5.00E-03**	**0.008**
cg06500161	21:43656587	*ABCG1*	4.39 (2.01)	3.00E-02	5.96 (2.43)	1.50E-02	4.76 (1.56)	2.00E-03	0.010

Table S2. Eight CpG sites associated with AUC in responses to a high-fat meal in the discovery, replication, and full samples of GOLDN

			Discovery (n=653)		Replication (n=326)		Full sample (n=979)		
Marker	Chr:position	Gene	beta (se)	*P*-Value	beta (se)	*P*-Value	beta (se)	*P*-Value	AUC variance explained
cg16464007	3:188002729	*LPP*	10.43 (2.90)	3.39E-04	18.76 (4.12)	7.57E-06	12.81 (2.32)	4.50E-08	0.030
cg00574958	11:68607622	*CPT1A*	-33.24 (4.91)	**3.02E-11**	-50.84 (6.27)	**1.18E-14**	-38.50 (3.77)	2.69E-23	0.097
cg09737197	11:68607675	*CPT1A*	-12.12 (3.45)	4.71E-04	-25.87 (4.75)	1.05E-07	-16.79 (2.75)	1.39E-09	0.037
cg17058475	11:68607737	*CPT1A*	-19.93 (3.57)	**3.58E-08**	-33.53 (5.01)	**1.01E-10**	-23.86 (2.84)	1.39E-16	0.068
cg01082498	11:68608225	*CPT1A*	-38.42 (9.41)	4.98E-05	-61.31 (12.61)	1.83E-06	-43.83 (7.34)	3.33E-09	0.036
cg12556569	11:116664039	*APOA5*	3.41 (0.63)	**9.52E-08**	2.61 (0.92)	**4.97E-03**	2.94 (0.52)	2.30E-08	0.032
cg11024682	17:17730094	*SREBF1*	25.41 (4.64)	**6.10E-08**	16.77 (6.03)	**5.74E-03**	20.64 (3.63)	1.68E-08	0.032
cg06500161	21:43656587	*ABCG1*	16.47 (3.53)	3.83E-06	18.35 (4.98)	2.70E-04	16.59 (2.80)	4.25E-09	0.035

Table S3. Pearson's correlation coefficients (the first panel)and P-values (the second panel) between eight AUC-associated methylation sites and four SNPs (n=707)

Gene	Chr:position	Marker	rs3135506	rs662799	rs964184X	rs1024369	cg1646400	cg0057495	cg0973719	cg1705847	cg0108249	cg1255656	cg1102468	cg06500161
APOA5	11:116791691	rs3135506	1.000	-0.085	-0.642	0.071	-0.008	-0.039	-0.008	-0.023	-0.051	0.457	0.042	0.039
APOA5	11:116792991	rs662799	-0.085	1.000	-0.629	-0.011	0.016	-0.068	-0.057	-0.046	-0.008	0.480	-0.004	-0.022
near *APOA5*	11:116778201	rs964184	-0.642	-0.629	1.000	-0.014	0.017	0.077	0.031	0.043	0.041	-0.689	-0.044	-0.025
	7:69439901	rs10243693	0.071	-0.011	-0.014	1.000	0.038	-0.012	-0.034	-0.005	-0.045	-0.033	-0.014	-0.022
LPP	3:188002729	cg16464007	-0.008	0.016	0.017	0.038	1.000	-0.423	-0.634	-0.562	-0.455	0.041	-0.496	-0.417
CPT1A	11:68607622	cg00574958	-0.039	-0.068	0.077	-0.012	-0.423	1.000	0.701	0.819	0.626	-0.086	0.323	0.236
CPT1A	11:68607675	cg09737197	-0.008	-0.057	0.031	-0.034	-0.634	0.701	1.000	0.836	0.710	-0.063	0.653	0.378
CPT1A	11:68607737	cg17058475	-0.023	-0.046	0.043	-0.005	-0.562	0.819	0.836	1.000	0.724	-0.055	0.503	0.298
CPT1A	11:68608225	cg01082498	-0.051	-0.008	0.041	-0.045	-0.455	0.626	0.710	0.724	1.000	-0.016	0.501	0.341
APOA5	11:116664039	cg12556569	0.457	0.480	-0.689	-0.033	0.041	-0.086	-0.063	-0.055	-0.016	1.000	0.026	-0.014
SREBF1	17:17730094	cg11024682	0.042	-0.004	-0.044	-0.014	-0.496	0.323	0.653	0.503	0.501	0.026	1.000	0.449
ABCG1	21:43656587	cg06500161	0.039	-0.022	-0.025	-0.022	-0.417	0.236	0.378	0.298	0.341	-0.014	0.449	1.000

Gene	Chr:position	Marker	rs3135506	rs662799	rs964184	rs10243693	cg16464007	cg00574958	cg09737197	cg17058475	cg01082498	cg12556569	cg11024682	cg06500161
APOA5	11:116791691	rs3135506		2.34E-02	3.58E-83	5.86E-02	8.30E-01	2.95E-01	8.27E-01	5.43E-01	1.74E-01	1.06E-37	2.62E-01	3.06E-01
APOA5	11:116792991	rs662799	2.34E-02		9.90E-79	7.65E-01	6.77E-01	7.23E-02	1.30E-01	2.18E-01	8.30E-01	6.37E-42	9.24E-01	5.63E-01
near *APOA5*	11:116778201	rs964184	3.58E-83	9.90E-79		7.17E-01	6.43E-01	4.17E-02	4.17E-01	2.59E-01	2.75E-01	2.71E-100	2.40E-01	5.00E-01
	7:69439901	rs10243693	5.86E-02	7.65E-01	7.17E-01		3.13E-01	7.45E-01	3.66E-01	8.97E-01	2.33E-01	3.84E-01	7.01E-01	5.56E-01
LPP	3:188002729	cg16464007	8.30E-01	6.77E-01	6.43E-01	3.13E-01		4.14E-32	6.83E-81	4.40E-60	2.60E-37	2.82E-01	3.37E-45	3.78E-31
CPT1A	11:68607622	cg00574958	2.95E-01	7.23E-02	4.17E-02	7.45E-01	4.14E-32		1.04E-105	2.14E-172	7.35E-78	2.19E-02	1.29E-18	2.08E-10
CPT1A	11:68607675	cg09737197	8.27E-01	1.30E-01	4.17E-01	3.66E-01	6.83E-81	1.04E-105		1.32E-185	3.42E-109	9.18E-02	2.85E-87	2.18E-25
CPT1A	11:68607737	cg17058475	5.43E-01	2.18E-01	2.59E-01	8.97E-01	4.40E-60	2.14E-172	1.32E-185		1.99E-115	1.43E-01	1.52E-46	5.60E-16
CPT1A	11:68608225	cg01082498	1.74E-01	8.30E-01	2.75E-01	2.33E-01	2.60E-37	7.35E-78	3.42E-109	1.99E-115		6.74E-01	4.37E-46	1.04E-20
APOA5	11:116664039	cg12556569	1.06E-37	6.37E-42	2.71E-100	3.84E-01	2.82E-01	2.19E-02	9.18E-02	1.43E-01	6.74E-01		4.90E-01	7.07E-01
SREBF1	17:17730094	cg11024682	2.62E-01	9.24E-01	2.40E-01	7.01E-01	3.37E-45	1.29E-18	2.85E-87	1.52E-46	4.37E-46	4.90E-01		2.17E-36
ABCG1	21:43656587	cg06500161	3.06E-01	5.63E-01	5.00E-01	5.56E-01	3.78E-31	2.08E-10	2.18E-25	5.60E-16	1.04E-20	7.07E-01	2.17E-36	

Table S4. Eight CpG sites associated with AUC and TG (log10) in response to a high-fat meal in the full samples of GOLDN

			AUC (n=979)				TG(log10) (n=979)		
Marker	Chr:position	Gene	beta (se)	*P*-Value	AUC variance explained		beta (se)	*P*-Value	AUC variance explained
cg16464007	3:188002729	*LPP*	12.81 (2.32)	4.50E-08	0.030		1.10 (0.18)	4.41E-09	0.035
cg00574958	11:68607622	*CPT1A*	-38.50 (3.77)	2.69E-23	0.097		-3.04 (0.3)	8.84E-23	0.095
cg09737197	11:68607675	*CPT1A*	-16.79 (2.75)	1.39E-09	0.037		-1.34 (0.22)	1.46E-09	0.037
cg17058475	11:68607737	*CPT1A*	-23.86 (2.84)	1.39E-16	0.068		-1.85 (0.23)	8.95E-16	0.065
cg01082498	11:68608225	*CPT1A*	-43.83 (7.34)	3.33E-09	0.036		-3.62 (0.59)	9.84E-10	0.038
cg12556569	11:116664039	*APOA5*	2.94 (0.52)	2.30E-08	0.032		0.22 (0.04)	1.47E-07	0.028
cg11024682	17:17730094	*SREBF1*	20.64 (3.63)	1.68E-08	0.032		1.53 (0.29)	1.66E-07	0.032
cg06500161	21:43656587	*ABCG1*	16.59 (2.80)	4.25E-09	0.035		1.27 (0.22)	1.79E-08	0.032

Table S5. 71 SNPs of the *APOA5* region on the chromosome 11 associated with TG postprandial responses (n=707)							
SNP	Chr	Position	Gene	P-Value	Negative log10_pvalue	Regression Beta	Beta Standard Error
rs964184	11	116648917	*ZNF259*	3.62E-09	8.441	-1.499	0.251
rs11823543	11	116649135	*ZNF259*	8.63E-06	5.064	1.610	0.359
rs17120029	11	116650118	*ZNF259*	1.53E-05	4.815	-1.514	0.348
rs12286037	11	116652207	*ZNF259*	1.53E-05	4.815	-1.514	0.348
rs12285095	11	116658031	*ZNF259*	1.60E-05	4.796	1.502	0.346
rs12294259	11	116637146	*BUD13*	3.76E-05	4.424	-1.433	0.345
rs11820589	11	116633862	*BUD13*	4.50E-05	4.347	1.413	0.344
rs10488699	11	116632500	*BUD13*	6.31E-05	4.200	-1.374	0.341
rs11825181	11	116626258	*BUD13*	1.52E-04	3.818	1.283	0.337
rs12292921	11	116621963	*BUD13*	1.53E-04	3.816	1.282	0.337
rs662799	11	116663707	*APOA5*	2.21E-04	3.656	-1.340	0.361
rs651821	11	116662579	*APOA5*	3.01E-04	3.522	1.304	0.359
rs664059	11	116642137	*BUD13*	3.67E-04	3.435	0.601	0.168
rs2266788	11	116660686	*APOA5*	3.90E-04	3.409	-1.189	0.334
rs2075290	11	116653296	*ZNF259*	3.92E-04	3.406	1.188	0.334
rs3825041	11	116631707	*BUD13*	3.96E-04	3.403	-1.188	0.334
rs2160669	11	116647607	*?*	3.96E-04	3.402	1.187	0.334
rs10790162	11	116639104	*BUD13*	3.96E-04	3.402	1.187	0.334
rs7930786	11	116624727	*BUD13*	3.97E-04	3.402	1.188	0.334
rs6589564	11	116624153	*BUD13*	4.43E-04	3.354	1.164	0.330
rs6589566	11	116652423	*ZNF259*	8.47E-04	3.072	-1.157	0.345
rs6589565	11	116640237	*BUD13*	8.72E-04	3.060	1.040	0.311
rs6589567	11	116670676	*?*	1.36E-03	2.865	0.862	0.268
rs180326	11	116624703	*BUD13*	2.71E-03	2.567	0.546	0.182
rs180327	11	116623659	*BUD13*	3.46E-03	2.460	0.531	0.181
rs5110	11	116691634	*APOA4*	4.28E-03	2.369	1.248	0.435
rs603446	11	116654435	*ZNF259*	1.05E-02	1.981	0.429	0.167
rs918143	11	116630600	*BUD13*	1.44E-02	1.843	0.410	0.167
rs3741300	11	116631690	*BUD13*	1.75E-02	1.758	-0.399	0.167
rs3741301	11	116631391	*BUD13*	1.75E-02	1.758	-0.399	0.167
rs2849179	11	116620796	*BUD13*	1.76E-02	1.754	0.398	0.167
rs180340	11	116616402	*?*	1.77E-02	1.753	-0.398	0.167

rs2187126	11	116635784	*BUD13*	4.59E-02	1.338	0.712	0.356
rs10488698	11	116633947	*BUD13*	4.59E-02	1.338	-0.712	0.356
rs17519093	11	116629905	*BUD13*	5.04E-02	1.297	-0.700	0.357
rs17519079	11	116629153	*BUD13*	5.46E-02	1.263	-0.690	0.359
rs6589568	11	116670738	*?*	5.66E-02	1.247	-0.459	0.240
rs17440396	11	116621770	*BUD13*	5.72E-02	1.243	-0.681	0.358
rs17518841	11	116615244	*?*	5.81E-02	1.236	-0.679	0.358
rs623908	11	116640368	*BUD13*	7.50E-02	1.125	-0.303	0.170
rs610675	11	116640094	*BUD13*	7.67E-02	1.115	0.302	0.170
rs2075295	11	116628401	*BUD13*	9.93E-02	1.003	0.311	0.189
rs1729410	11	116665661	*?*	2.70E-01	0.568	-0.203	0.184
rs180325	11	116625294	*BUD13*	2.79E-01	0.555	-0.228	0.211
rs180324	11	116625332	*BUD13*	2.80E-01	0.553	-0.228	0.211
rs4938310	11	116629949	*BUD13*	2.88E-01	0.540	0.224	0.211
rs7942054	11	116632975	*BUD13*	3.36E-01	0.473	-0.206	0.214
rs1263173	11	116681008	*?*	3.89E-01	0.410	-0.166	0.193
rs11216129	11	116620256	*BUD13*	3.93E-01	0.406	0.216	0.252
rs12421652	11	116625875	*BUD13*	3.93E-01	0.405	-0.216	0.252
rs11216126	11	116617240	*?*	3.99E-01	0.399	-0.213	0.252
rs11216131	11	116633580	*BUD13*	4.07E-01	0.390	-1.145	1.379
rs1263167	11	116677723	*?*	4.34E-01	0.363	0.194	0.248
rs675	11	116691675	*APOA4*	4.40E-01	0.356	-0.192	0.248
rs1263149	11	116639941	*BUD13*	4.51E-01	0.346	-0.126	0.167
rs2075291	11	116661392	*APOA5*	4.85E-01	0.315	-0.727	1.040
rs918144	11	116633825	*BUD13*	5.34E-01	0.272	-0.104	0.167
rs619054	11	116660813	*APOA5*	5.60E-01	0.252	-0.117	0.200
rs4417316	11	116652301	*ZNF259*	6.10E-01	0.215	-0.154	0.302
rs1729409	11	116673768	*?*	6.26E-01	0.203	0.090	0.184
rs11602073	11	116646858	*?*	6.27E-01	0.203	-0.141	0.290
rs1942478	11	116651463	*ZNF259*	6.32E-01	0.199	0.145	0.302
rs618923	11	116654159	*ZNF259*	7.37E-01	0.132	0.067	0.200
rs11556024	11	116619051	*BUD13*	7.41E-01	0.130	-0.340	1.030
rs1729407	11	116677370	*?*	8.06E-01	0.094	0.044	0.179
rs633389	11	116667337	*?*	8.23E-01	0.084	0.162	0.727

rs17120035	11	116663851	*APOA5*	8.55E-01	0.068	-0.061	0.336
rs11600380	11	116670182	*?*	8.62E-01	0.065	0.058	0.332
rs2075294	11	116658122	*ZNF259*	8.84E-01	0.053	0.118	0.812
rs9804646	11	116665079	*?*	9.63E-01	0.017	-0.016	0.332
rs11216137	11	116669828	*?*	9.63E-01	0.016	0.015	0.330
?=no gene known.							

Table S6. Pearson's correlation coefficients (the left panel) and P-values (the right panel) between fasting triglyceride (at baseline) and postprandial TG response measures (n=979)

Variable	Triglyceride	TG uptake slope	TG clearance slope	TG area under the curve increase	TG Area under the whole curve		Triglyceride	TG uptake slope	TG clearance slope	TG area under the curve increase	TG Area under the whole curve
Triglyceride (fasting), mg/dL	1.000	-0.103	0.149	-0.036	0.853			1.22E-03	2.85E-06	2.57E-01	9.58E-279
TG uptake slope	-0.103	1.000	0.844	0.989	0.157		1.22E-03		1.05E-266	0.00E+00	8.39E-07
TG clearance slope	0.149	0.844	1.000	0.915	0.393		2.85E-06	1.05E-266		0.00E+00	2.00E-37
TG area under the curve increase	-0.036	0.989	0.915	1.000	0.227		2.57E-01	0.00E+00	0.00E+00		5.91E-13
TG Area under the whole curve	0.853	0.157	0.393	0.227	1.000		9.58E-279	8.39E-07	2.00E-37	5.91E-13	
